# The Relationships Between Ethical Sensitivity, Ethical Decision-Making Ability, and Ethical Conflict Among ICU Nurses: A Structural Equation Model

**DOI:** 10.1155/jonm/7756343

**Published:** 2025-04-03

**Authors:** Qingyun He, Chunmei Huang, Zhixian Feng, Huajuan Shen

**Affiliations:** ^1^Department of Emergency Intensive Care Unit, Tongde Hospital of Zhejiang Province Affiliated to Zhejiang Chinese Medical University, Hangzhou, Zhejiang, China; ^2^Hospital-Acquired Infection Control Department, Tongde Hospital of Zhejiang Province Affiliated to Zhejiang Chinese Medical University, Hangzhou, Zhejiang, China; ^3^Nursing Department, Shulan(HangZhou) Hospital, Hangzhou, Zhejian, China; ^4^Nursing Department, Zhejiang Provincial People's Hospital, Hangzhou, Zhejiang, China

**Keywords:** ethical conflict, ethical decision-making ability, ethical sensitivity, intensive care unit, nurse

## Abstract

**Background:** Ethical conflict in nursing is a common phenomenon in intensive care units (ICUs). Nurses' ethical sensitivity forms the basis for identifying ethical conflicts. Ethical decision-making abilities are closely related to ethical conflict. However, there are currently no reports on the pathways between ethical sensitivity, decision-making ability, and conflicts among ICU nurses.

**Purpose:** Based on the cognitive-behavioral theory, a structural equation model was developed to quantitatively analyze the relationships between ICU nurses' ethical sensitivity, decision-making ability, and conflicts.

**Methods:** A cross-sectional survey was conducted involving ICU nurses from six general hospitals in China from May to July 2024, using the General Information Questionnaire, Ethical Conflict Nursing Questionnaire–Critical Care Version (ECNQ-CCV), Chinese Moral Sensitivity Questionnaire–Revised Version (MSQ-R-CV, including the dimensions of moral responsibility and strength and sense of moral burden), and Chinese version of judgment about nursing decision (JAND-CE). Descriptive analyses were conducted with SPSS 25.0, and a structural equation model (using Amos 26.0) was performed to identify path relationships between the variables.

**Results:** The constructed model demonstrated a strong overall fit, and there were significant correlations between ethical sensitivity, decision-making ability, and conflicts among Chinese ICU nurses (*p* < 0.05). The values of path coefficients showed that moral responsibility and strength have a positive association with JAND-CE (*β* = 0.263, *p* < 0.05) and negative association with ECNQ-CCV (*β* = −0.246, *p* < 0.05). Moreover, sense of moral burden has a negative association with JAND-CE (*β* = −0.353, *p* < 0.05) and positive association with ECNQ-CCV (*β* = 0.232, *p* < 0.05). Further, JAND-CE has a negative association with ECNQ-CCV (*β* = −0.183, *p* < 0.05). This study conducted mediation analysis by examining the indirect path between moral responsibility and strength, sense of moral burden, and ECNQ-CCV via JAND-CE, whereby the beta coefficients of independent mediating and mediating-dependent variables were multiplied. The indirect path between moral responsibility and strength and ECNQ-CCV through JAND-CE was significant (i.e. indirect path (0.263 × (−0.183)) = −0.048, *p* < 0.05, LL = −0.608, UL = −0.07), and the indirect path between sense of moral burden and ECNQ-CCV through JAND-CE was significant (indirect path ((-0.353) × (−0.183)) = 0.065, *p* < 0.05, LL = 0.082, UL = 0.758) and did not contain a zero value between lower and upper boundaries.

**Conclusions:** This study reveals the dual-path mechanism of moral responsibility and strength and sense of moral burden on ethical conflicts through structural equation modeling, emphasizing the mediating pivotal role of ethical decision-making ability. The research findings provide a theoretical basis for the refinement of moral capacity cultivation systems, while also warning of the potential negative impacts of moral burden.

**Implications for nursing managers:** Nursing managers should dynamically evaluate ICU nurses' ethical sensitivity and decision-making abilities to provide a reference for implementing individualized ethical conflict intervention measures.

## 1. Introduction

Ethical conflict in nursing is a common phenomenon that involves responses to ethically challenging situations related to conflicting ideals and competing interests among different stakeholders, as well as unclear requirements in nursing practice [1]. It includes moral uncertainty, dilemma, distress, outrage, indifference, and wellbeing [2]. The continued existence of ethical conflict may result in adverse consequences to the physiological and psychological health of nurses [3], a decline in nursing quality, threats to patient health and safety, and even significant losses in organizational productivity and economy [4, 5]. Intensive care unit (ICU) nurses lack autonomy in clinical decision-making in complex nursing practice environments and often face ethical conflicts [6, 7].

ICU nurses alleviate ethical conflicts based on their own experiences, employing strategies such as ignoring ethical issues in the workplace, seeking ways to express emotions, and putting themselves in others' shoes [8]. The perception of ethical issues among nurses affects their ethical behavior, and the levels of ethical conflict also differ [9, 10]. Therefore, further exploration of how ICU nurses adapt to ethical conflicts is crucial for developing personalized intervention measures. Nevertheless, previous studies have concentrated mainly on exploring the pathways of ethical sensitivity, ethical decision-making ability, and ethical conflicts among nursing students or interns, and there are currently no such reports on ICU nurses [11].

Ethical sensitivity refers to nurses' understanding of ethical values in clinical contexts and self-awareness of their roles and responsibilities. This is a fundamental element of nurses' ethical behavior [12]. Therefore, it is considered a key factor at the individual level for recognizing ethical conflicts [13]. Lower ethical sensitivity is associated with fewer ethical conflicts; if nurses cannot recognize ethical issues, they may easily ignore them [14]. Nurses' awareness of ethical issues is related to their ethics training experience. Nurses who lack theoretical knowledge and experience related to ethics have lower ethical sensitivity [15]. Thus, ethics education and training are necessary for nurses to acquire ethical sensitivity [16]. Simultaneously, providing ethical education and training can enhance nurses' ethical decision-making abilities, which is beneficial for generating positive psychological reactions and improving ethical sensitivity [17].

Ethical decision-making ability is a concentrated manifestation of nurses' behavioral responses throughout the process of ethical decision-making, including the ability to describe the ethical situation they are in, define problems from an ethical perspective, analyze alternative methods, envisage possible outcomes, and choose the best course of action [18]. Ethical cognition is related to ethical behavioral responses. If nurses cannot perceive ethical issues, their decision-making abilities will not matter [9, 19]. Accordingly, ethical sensitivity is a precondition for ethical decision-making [20]. Nurses with lower educational levels and a lack of ethical knowledge are more prone to ethical conflicts [5, 21]. However, nurses who have participated in ethics training, possess theoretical knowledge of ethics, are good at identifying ethical issues, and know the correct ethical behavior, but are unable to make decisions on the best course of action, are also prone to ethical conflicts [22, 23].

Ethical conflict is related to ethical cognition (e.g., awareness of moral values) and informational perspectives (e.g., define problems from an ethical perspective) in ethical practice [24]. This aligns with the cognitive-behavioral theory [25], which posits that automatic thoughts (immediate moral intuitions, e.g., gut reactions to ethical violations), conditional assumptions (context-dependent assumptions, e.g., “prioritizing ethics compromises operational efficiency”), and core beliefs (foundational self-perceptions, e.g., ethical self-efficacy in moral reasoning) collectively constitute an individual's cognitive architecture, shaping behavioral patterns and decision-making processes, and continuously influencing individuals' behaviors and emotional responses. Adaptive core beliefs (e.g., strong self-efficacy) foster constructive behavioral outcomes by enhancing cognitive flexibility in problem-solving and facilitating emotional regulation. In organizational contexts, ethical leadership amplifies this mechanism by mitigating workplace stressors, such as emotional exhaustion and anxiety stemming from perceived injustice, thereby freeing cognitive resources for employees to engage in innovation-driven activities [26]. Furthermore, employees with elevated knowledge reserves demonstrate greater capacity to operationalize institutional support into tangible innovation practices through enhanced metacognitive processing [27]. Conversely, maladaptive cognitive patterns, such as irrational automatic thoughts manifesting as moral hypervigilance, may engender counterproductive behaviors through dual pathways of cognitive distortion (e.g., catastrophizing ethical dilemmas) and psychological resource depletion.

Structural equation modeling (SEM) integrates two statistical methods: factor and path analyses. This confirmatory method requires theoretical or empirical support to construct a theoretically guided hypothesis model. SEM has been applied to analyze various factors affecting behavioral changes and study their action pathways. Raylu et al. [28] used SEM to test the effectiveness of the hypothesized approach in the cognitive-behavioral theory for gambling behavior. Based on SEM, Fitrianie et al. [29] explored the differences between behavioral intention applications and usage behavior when using cognitive behavior therapy to treat insomnia.

Grounded in the cognitive-behavioral theory, this research examined the connections among ethical sensitivity, decision-making ability, and conflict among ICU nurses. A structural equation model was constructed to explore the path relationship between ethical conflict and various variables, with the results providing targeted suggestions for alleviating ethical conflicts among ICU nurses. The hypotheses for the research were as follows:

  H1: Ethical sensitivity positively correlates with ethical conflict.
  H2: Ethical decision-making ability negatively correlates with ethical conflict.
  H3: Ethical sensitivity positively correlates with ethical decision-making ability.
  H4: Ethical decision-making ability mediates between ethical sensitivity and ethical conflict.

**Figure 1:**
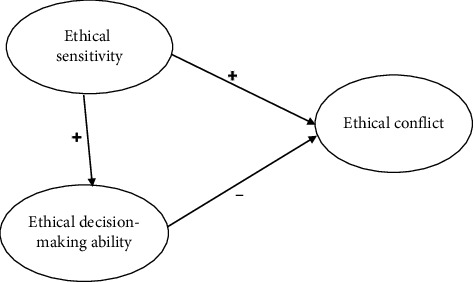
Hypothesized theoretical model.

The hypothesized theoretical model is shown in Figure 1.

## 2. Materials and Methods

### 2.1. Study Design and Participants

This research conducted a cross-sectional survey using a convenience sampling method to select ICU nurses from six comprehensive hospitals in Zhejiang, Guangdong, and Guangxi provinces, China, from May to July 2024. The inclusion criteria were as follows: (a) age ≥ 18 years; (b) six months or more of ICU work experience; and (c) voluntary participation. The exclusion criteria were as follows: (a) left for 1 month or more without returning to work; (b) trainee, intern, or rotation nurses; and (c) severe physical and mental disorders or family and social issues (significant problems that negatively impact individuals, families, or society as a whole, such as domestic violence, divorce or separation, poverty, substance abuse or addiction, mental health issues, child abuse or neglect, aging and elder care challenges, unemployment or job instability, social exclusion or discrimination, education inequality, etc.). The sample capacity was confirmed as 10 participants for each variable, assuming an attrition rate of 20% [30]. Fourteen population characteristics and four scale-related dimensions were considered as independent variables, requiring at least (14 + 4) × 10 × (1 + 20%) = 216 participants.

### 2.2. Measurements

#### 2.2.1. Participant Characteristics

Based on prior investigations [1, 22], a custom-tailored general information survey was used to gather participants' general information. The questions included sex, age, having religious beliefs, having siblings, marital status, education level, type of employment, years of critical care experience, professional title, years of nursing experience, type of ICU, job level, ethics-related training, and hospital level.

#### 2.2.2. Ethical Conflict Nursing Questionnaire-Critical Care Version (ECNQ-CCV)

Ethical conflict was evaluated using the unidimensional ECNQ-CCV [31]. The questionnaire consists of 19 critical care scenarios, each with three questions to assess the frequency, intensity, and type of ethical conflict. Frequency was scored using a six-point Likert scale from 0 to 5, ranging from “never” to “at least once a week.” Intensity was scored using a 5-point Likert scoring system, ranging from “nonexistent” to “a serious issue,” scored from 1 to 5. The score for each scenario was the product of the frequency and intensity scores, with the total score ranging from 0 to 475. The higher the score, the more severe the nurses' ethical conflict. The Chinese version of the questionnaire has good internal consistency, with a Cronbach's alpha of 0.902 and a test-retest reliability of 0.757 [32]. In this study, Cronbach's alpha was 0.988.

#### 2.2.3. Chinese Moral Sensitivity Questionnaire–Revised Version (MSQ-R-CV)

Ethical sensitivity is interchangeable with moral sensitivity, as professional behavior is typically guided by ethical standards that have gradually replaced moral sensitivity [33]. Ethical sensitivity was assessed using the MSQ-R-CV [34]. The questionnaire consists of nine items in two dimensions: moral responsibility and strength (five items) and moral burden (four items). A six-point Likert scoring method was adopted (1 = strongly disagree and 6 = strongly agree). The total score was the sum of the scores for each item, ranging from 9 to 54 points. The higher the score, the stronger the ethical sensitivity. The MSQ-R-CV has good reliability and validity with Cronbach's alpha of 0.82. In this study, Cronbach's alpha of the first part (moral responsibility and strength) was 0.848, and that of the second part (moral burden) was 0.862.

#### 2.2.4. Chinese Version of Judgment About Nursing Decision (JAND-CE)

Ethical decision-making ability was evaluated using the Chinese version of the judgment about nursing decision (JAND-CE) [35]. This instrument includes six stories requiring clinical decision-making and lists six to seven problems for nurses to solve after each story, totaling 37 questions (i.e., 37 items). The respondents used a five-point Likert grading system (5 = strongly agree, 4 = agree, 3 = neutral, 2 = disagree, and 1 = strongly disagree) to rate each item in Columns *A* and *B* according to the following requirements: Column *A*: the behavior that nurses should perform in an ideal situation without any restrictions, classified as an ethical choice; Column *B*: owing to various limiting factors, the actions that nurses in your hospital may take in reality, classified as ethical actions. Participants were asked to briefly explain the reasons for adopting these behaviors in Column *C*. The ethical choices in Column A were divided into three categories: “not recommended” (6 items), “ambiguous” (8 items), and “recommended” (23 items); the answers were not given to the respondents. The total score in Column A ranged from 55 to 185 points, with higher scores indicating stronger ethical decision-making abilities. Among them, the “recommended” items were scored as 5, 4, 3, 2, and one point; items that were not recommended were scored on a scale of 1, 2, 3, 4, and five points; ambiguous items were scored on a scale of 3, 4, 5, 4, and three points. The highest score (Columns *A* + *B*) was 370 (highest nursing ethics decision-making ability), and the lowest score was 110 (lowest decision-making ability). A score greater than 296 points (80% of the total score) was defined as a high level of ethical decision-making ability, a score of 222–296 was considered moderate (accounting for 60% of the total score), and a score less than 222 was considered low (below 60% of the total score). The Chinese version of the JAND showed good test-retest reliability (*r* = 0745 > 0.7, *p*  < 0.01) and expert content validity (I-CV1 = 0.982). In this study, Cronbach's alpha was 0.878.

### 2.3. Data Collection

The research team held an online project launch meeting in which the researchers described the background, objectives, and significance of the study. Permission was obtained from the nursing managers in the hospitals. Data collection methods were standardized during the meeting, with a unified guiding language and instructions for completing the electronic questionnaires. Before the formal investigation, a presurvey was conducted with 50 ICU nurses [36]. Based on the feedback from the presurvey, we revised the questionnaire to enhance the readability of its content. Each hospital appointed a project leader and two investigators to administer the questionnaires. The surveys were distributed using the Question Star platform, and participants were required to answer every question to ensure that no data were missing. The questionnaires were shared with participants via QR codes, which were disseminated through hospital communication channels such as internal emails, staff meetings, or hospital bulletin boards. The team collected 330 questionnaires and excluded those that were completed within one minute or had completely identical answers. Finally, 328 questionnaires were analyzed to meet the required sample size.

### 2.4. Statistical Analysis

Data were analyzed using SPSS 25.0. Descriptive analysis, a normality test, and Pearson's correlation analysis were performed, with *p* < 0.05 indicating statistically significant differences. AMOS26.0 was used for confirmatory factor analysis (CFA), convergence validity, combination validity of various dimensions of the scales, discriminant validity, model fit, path relationship hypothesis testing, and constructing the model. The model path effect values were validated using the bootstrap method. All tests indicated statistically significant differences at *p* < 0.05 (two-tailed).

### 2.5. Ethical Considerations

This study was conducted following the principles of the Declaration of Helsinki. Approval was obtained from the Medical Ethics Committee of the Tongde Hospital of Zhejiang Province (approval number: Zhentong de Leng Review 2024 study no. 083-JY). All research participants were informed that their participation implied informed consent and were exempted from signing the informed consent form after obtaining approval from the ethics committee. All respondents' information was kept confidential, and individuals reserved the right to withdraw from the investigation at any time.

## 3. Results

### 3.1. Participants' Characteristics

Among the 328 ICU nurses, 75% were female, with 72.56% aged between 26 and 40 years. A total of 87.5% had a bachelor's degree or higher, and 71.04% had received ethics training. Specific patient characteristics are listed in Table 1.

### 3.2. Reliability Analysis of the MSQ-R-CV, JAND-CE, and ECNQ-CCV for ICU Nurses

Reliability analysis was performed in the form of a scale in this study. Therefore, verifying the data quality of the measurement results was an essential prerequisite for ensuring the significance of the subsequent analysis. Cronbach's alpha ranges from 0 to 1, and the higher the coefficient value, the higher the reliability. A reliability coefficient below 0.6 is considered unreliable and requires a redesigning of the questionnaire or a fresh attempt to collect data and conduct further analyses. A reliability coefficient between 0.6 and 0.7 is considered reliable, between 0.7 and 0.8 is relatively reliable, between 0.8 and 0.9 is very reliable, and between 0.9 to 1 is very reliable.

Table 2 presents the results of reliability analyses. The reliability coefficient for each scale was > 0.8, indicating that the scales used in this study had good internal consistency and reliability.

### 3.3. Validity Analysis of the MSQ-R-CV, JAND-CE, and ECNQ-CCV for ICU Nurses

#### 3.3.1. Validation Factor Analysis

According to the model adaptation test results in Table 3, the CMIN/DF (chi-square degree of freedom ratio) of the MSQ-R-CV, JAND-CE, and ECNQ-CCV were all within the range of one to three, and the root mean square error of approximation (RMSEA) was within the good and excellent range. The incremental fit index (IFI), Tucker–Lewis index (TLI), and comparative fit index (CFI) test results reached a significant level of 0.9 or above. Therefore, based on the comprehensive analyses, the CFA models of the MSQ-R-CV, JAND-CE, and ECNQ-CCV had a good fit.

#### 3.3.2. Convergent Validity and Combinatorial Validity Tests

The convergent validity and combination reliability of each dimension of the scales were further tested under the premise that the CFA models of the MSQ-R-CV, JAND-CE, and ECNQ-CCV had good adaptability. The verification process calculated the standardized factor loadings of each measurement item in the corresponding dimension using the established CFA model. The convergent validity and combination reliability values of each dimension were then calculated using the average variance extracted (AVE) and critical rantion (CR) formulas. According to the standard, an AVE value of 0.5 or above and a CR value of 0.7 or above indicate good convergence validity and combination reliability, respectively.

According to the analysis results in Table 4, in the validity tests of the MSQ-R-CV, JAND-CE, and ECNQ-CCV, the AVE values of each dimension were 0.5 or above, and the CR values were 0.7 or above. Overall, it can be concluded that all dimensions had good convergent validity and combination reliability.

#### 3.3.3. Discriminant Validity of the MSQ-R-CV

According to the analysis results in Table 5, in the discriminant validity test, the standardized correlation coefficients between each dimension were all smaller than the square root of the AVE values corresponding to each dimension, indicating good discriminant validity between each dimension.

### 3.4. Descriptive Statistics and Normality Tests

Table 6 presents the results of the statistical analysis and normality test for the factors in this study. A normality test for each measurement item was conducted using skewness and kurtosis values. According to Kline's [30] standard, if the absolute value of the skewness coefficient is within 3 and the absolute value of the kurtosis coefficient is within 8, the data are considered to meet the requirements of an approximate normal distribution. According to the results presented in Table 6, the absolute skewness and kurtosis coefficients for each measurement item in this study were within the standard range. Therefore, the data for each measurement parameter followed an approximately normal distribution.

### 3.5. Analysis of the Correlation Between Ethical Sensitivity, Ethical Decision-Making Ability, and Ethical Conflict Among ICU Nurses

Pearson's correlation analysis was performed to determine the correlations among variables. As shown in Table 7, there were significant correlations among the variables, and all results were significant at the 95% level. The correlation coefficients between moral responsibility and strength, ethical decision-making ability, and ethical conflict were all less than 0, while the correlation coefficient between moral burden and ethical conflict was greater than 0. Therefore, there was a negative correlation between moral responsibility and strength, ethical decision-making ability, and ethical conflict, and there was a positive correlation between moral burden and ethical conflict.

### 3.6. SEM

#### 3.6.1. Model Fitness Test for the Factors Influencing Ethical Conflict

According to the model fitness test results in Table 8, the CMIN/DF of the model of the factors influencing ethical conflict was 1.490, which was in the excellent range, and the RMSEA was 0.039, which was within the acceptable range. Additionally, the IFI, TLI, and CFI test results were excellent (> 0.9). Therefore, this analysis showed that the SEM was a good fit for analyzing the factors affecting ethical conflicts.

#### 3.6.2. Path Hypothesis Test Results for the Model of Factors Influencing Ethical Conflict

As indicated in Table 9, this study examined the structural model at 5000 bootstraps with 95% confidence interval. The values of path coefficients showed that moral responsibility and strength have a positive association with JAND-CE (*β* = 0.263, *p* < 0.05) and negative association with ECNQ-CCV (*β* = −0.246, *p* < 0.05). Moreover, sense of moral burden has a negative association with JAND-CE (*β* = −0.353, *p* < 0.05) and positive association with ECNQ-CCV (*β* = 0.232, *p* < 0.05). Further, JAND-CE has a negative association with ECNQ-CCV (*β* = −0.183, *p* < 0.05). This study conducted mediation analysis by examining the indirect path between moral responsibility and strength, sense of moral burden, and ECNQ-CCV via JAND-CE, whereby the beta coefficients of independent mediating and mediating-dependent variables were multiplied. The indirect path between moral responsibility and strength and ECNQ-CCV through JAND-CE was significant (i.e. indirect path (0.263 × (−0.183)) = −0.048, *p* < 0.05, LL = −0.608, UL = −0.07), and the indirect path between sense of moral burden and ECNQ-CCV through JAND-CE was significant (indirect path ((-0.353) × (−0.183)) = 0.065, *p* < 0.05, LL = 0.082, UL = 0.758) and did not contain a zero value between lower and upper boundaries.

**Figure 2:**
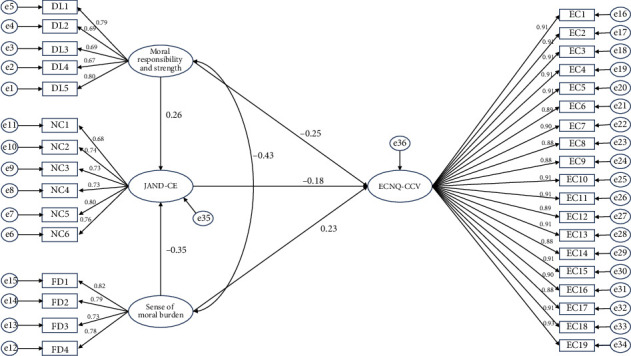
Standardized estimates of the relationships and effect sizes in the structural equation model. Abbreviations: DL, moral responsibility and strength; EC, ECNQ-CCV, Ethical Conflict Nursing Questionnaire–Critical Care Version; FD, sense of moral burden; NC, JAND-CE, Chinese version of judgment about nursing decision.

Figure 2 showcases the detailed relationships among observed, latent, exogenous, and endogenous variables.

## 4. Discussion

Based on the cognitive-behavioral theory, this study selected variables to explore the associations among ethical sensitivity, ethical decision-making ability, and ethical conflicts among Chinese ICU nurses and analyzed the relationship paths between these three variables. The results showed that the constructed SEM had good overall adaptability and that ethical sensitivity and decision-making ability had strong predictive and explanatory powers for ethical conflicts.

Ethical sensitivity is a multidimensional construct (e.g., moral responsibility and strength and sense of moral burden). Specifically, among the two dimensions of ethical sensitivity, moral responsibility and strength belong to the positive dimension, while the sense of moral burden belongs to the negative dimension [34]. By analyzing these dimensions separately, we can more accurately capture these differences and provide richer theoretical insights [30]. The results confirmed that nurses' moral responsibility and strength were positively related to ethical decision-making ability; conversely, a sense of moral burden was negatively related to ethical decision-making ability, which is consistent with the results of previous studies [37, 38]. Although no study has directly examined the relationship between ethical sensitivity and ethical conflict, this study found that moral responsibility and strength were negatively related to ethical conflict, and a sense of moral burden was positively related to ethical conflict. This further confirmed the relationship between ethical sensitivity and ethical dilemmas in previous studies [39] and aligned with the perspective in the cognitive-behavioral theory that “cognitive structures influence behavioral outcomes” [40]. Ethical decision-making ability was negatively related to ethical conflict, which confirms previous findings [41]. When facing ethical conflict, moral responsibility and strength could give nurses the courage to take ethical actions according to their own ethical decisions and enhance their sense of responsibility and mission toward their work. At that point, the level of ethical conflict was relatively low. Contrarily, as a negative dimension of ethical sensitivity, nurses with higher scores on the moral burden dimension were more likely to experience high levels of ethical conflict. Nursing managers should attach importance to the positive and negative dimensions of nurses' ethical sensitivity, provide guidance and education when necessary, and combine cognitive behavioral therapy (e.g., cognitive restructuring techniques) [42] with other psychological therapies (e.g., mindfulness-based stress reduction) [43] to help nurses form positive attitudes, strengthen their ethical decision-making abilities, and potentially address ethical conflicts.

Furthermore, this study elucidated the mediating role of ethical decision-making ability. The finding that moral responsibility and strength indirectly alleviated ethical conflicts through enhanced ethical decision-making aligns with cognitive-behavioral theory's proposition regarding “the regulatory effects of core beliefs on secondary cognitive hierarchies (conditional beliefs and automatic thoughts)” [40]. For instance, nurses with heightened moral responsibility were more likely to develop conditional beliefs such as “ethical practice does not excessively deplete resources”, consequently reducing emotional exhaustion. Conversely, sense of moral burden exacerbated ethical conflicts through compromised decision-making ability (e.g., decision fatigue), our understanding of the theory that “disruptive feedback loops between automatic thoughts and conditional beliefs” [40]. These findings underscored the criticality of emotion regulation in nursing ethics practice. Importantly, the results showed that solely enhancing ethical decision-making ability demonstrated limited efficacy in ethical conflict mitigation. Attention should be paid to the psychological burden of medical staff, avoiding overemphasizing “perfect morality” which can lead to a decline in decision-making ability.

While existing strategies, such as ethics empowerment programs [44, 45], clinical ethics consultation [46], and ethical climate cultivation, show promise [19], the unique stressors in ICU settings demand targeted interventions [47]. The significant role of individual differences in ethical conflict perception underscores the need to transition from standardized ethics training to personalized intervention paradigms. For nurses with high moral responsibility but a high burden, cognitive dissociation techniques in cognitive behavioral therapy (such as observational thinking rather than identification thinking) can be used to reduce the interference of moral burden on ethical decision-making ability. Nurses with low moral responsibility and strength can strengthen the direct transformation of moral beliefs into behavior through behavioral activation training (such as role-playing that simulates ethical conflict situations). Medical institutions can adopt a layered intervention strategy: primary prevention focuses on enhancing moral responsibility and strength (such as ethical narrative training) [48]; secondary buffering strengthens ethical decision-making capabilities with instrumental support (such as decision-making flowcharts); and the tertiary response establishes a dynamic monitoring and psychological counseling mechanism for moral burden. The organizational support is optimized, and a supportive environment is built simultaneously through systematic measures, such as ethical performance evaluation and conflict response teams [49].

## 5. Limitations

This study used SEM to investigate the relationship between ethical sensitivity, ethical decision-making ability, and ethical conflict among ICU nurses in China, providing valuable information for nursing administrators and individual nurses to mitigate ethical conflict. However, this was a cross-sectional study that did not reflect the long-term effects of ethical sensitivity and decision-making ability on ethical conflicts. Second, the relationship between ethical climate, healthcare collaboration, and ethical conflict has been demonstrated, but owing to research resource constraints, it was not included in this study; their interrelationships need to be explored in further depth. In future studies, researchers should collect data from a wider range of hospitals to determine whether the findings would differ in a broader and more representative sample.

## 6. Conclusions

Based on the cognitive-behavioral theory framework, this study constructed a SEM of ethical sensitivity, ethical decision-making ability, and ethical conflict among Chinese ICU nurses, which provides a new perspective for exploring individual-level factors and pathways that influence ethical conflict among nurses in the ICU. Ethical sensitivity and decision-making abilities interact to influence the level of ethical conflict. In clinical nursing practice, nursing managers should comprehensively assess nurses' ethical sensitivity, ethical decision-making ability, and ethical conflict levels before designing an intervention program for ethical conflicts in the ICU, considering the potential pathways between these variables and ethical conflict. Through consultation, education, and psychological support, the positive role of nurses' ethical sensitivity could be leveraged to improve their ethical decision-making abilities and help them cope with ethical conflicts.

## Figures and Tables

**Table 1:** Participants' characteristics (*n* = 328).

Variables	*n* (%)
Sex	
Male	82 (25.00%)
Female	246 (75.00%)
Age (years)	
18–25	72 (21.95%)
26–30	124 (37.80%)
31–40	114 (34.76%)
41–50	18 (5.49%)
Having religious beliefs	
Yes	16 (4.88%)
No	312 (95.12%)
Having siblings	
Yes	255 (77.74%)
No	73 (22.26%)
Marital status	
Married	166 (50.61%)
Unmarried	154 (46.95%)
Divorced or other	8 (2.44%)
Education level	
College or lower	41 (12.50%)
Baccalaureate	273 (83.23%)
Master or above	14 (4.27%)
Type of employment	
Public institutions	135 (41.16%)
Contract terms	157 (47.87%)
Personnel agency	36 (10.97%)
Years of critical care experience	
0.5–5	156 (47.56%)
6–10	108 (32.93%)
11–15	46 (14.02%)
15 or more	18 (5.49%)
Professional title	
Nurse	65 (19.82%)
Senior nurse	165 (50.30%)
Supervisor nurse	86 (26.22%)
Cochief nurse	12 (3.66%)
Years of nursing experience	
5 or less	122 (37.20%)
5–10	118 (35.98%)
11–15	55 (16.77%)
16–20	17 (5.18%)
20 or more	16 (4.88%)
Type of ICU	
General	263 (80.18%)
Emergency	47 (14.33%)
Other	18 (5.49%)
Job level	
N0	27 (8.23%)
N1	80 (24.39%)
N2	125 (38.11%)
N3	63 (19.21%)
N4	21 (6.40%)
Chief nurse	12 (3.66%)
Ethics-related training	
Yes	233 (71.04%)
No	95 (28.96%)
Hospital level	
Grade 3B	66 (20.12%)
Grade 3A	262 (79.88%)

*Note:* Public institution nurses, after being recruited by the government (such as the health bureau), are assigned to work in hospitals, and their salary, benefits, and retirement benefits are supported by the government; contract nurses sign labor contracts directly with the hospitals; personnel agency nurses sign contracts with a local talent exchange center or a personnel agency. Ethics-related training, nurses who have participated in ethics workshops, hospital-based ethics training, or nursing ethics courses at academic institutions are considered to have received ethics-related training.

**Table 2:** Reliability analysis among the study variables.

Variables	Cronbach's alpha	Number of items
Moral responsibility and strength	0.848	5
Sense of moral burden	0.862	4
JAND-CE	0.878	6
ECNQ-CCV	0.988	19

*Note:* JAND-CE, Chinese version of judgment about nursing decision.

Abbreviations: ECNQ-CCV, Ethical Conflict Nursing Questionnaire–Critical Care Version.

**Table 3:** Results of the model fit test for each scale.

Norm	Reference standard	Results		
		MSQ-R-CV	JAND-CE	ECNQ-CCV
CMIN/DF	1–3 as excellent and 3–5 as good	1.465	1.963	2.410
RMSEA	< 0.05 as excellent and < 0.08 as good	0.038	0.054	0.066
IFI	> 0.9 as excellent and > 0.8 as good	0.991	0.993	0.977
TLI	> 0.9 as excellent and > 0.8 as good	0.987	0.984	0.974
CFI	> 0.9 as excellent and > 0.8 as good	0.990	0.993	0.977

*Note:* CMIN/DF, chi-square degree of freedom ratio; MSQ-R-CV, Chinese Moral Sensitivity Questionnaire–Revised Version; JAND-CE, chinese version of judgment about nursing decision.

Abbreviations: CFI, comparative fit index; ECNQ-CCV, Ethical Conflict Nursing Questionnaire–Critical Care Version; IFI, incremental fit index; RMSEA, root mean square error of approximation; TLI, Tucker–Lewis index.

**Table 4:** Convergent validity and combined reliability tests for each dimension.

Path relationship			Estimate	AVE	CR
DL1	<---	Moral responsibility and strength	0.786	0.532	0.850
DL2	<---	Moral responsibility and strength	0.688		
DL3	<---	Moral responsibility and strength	0.690		
DL4	<---	Moral responsibility and strength	0.674		
DL5	<---	Moral responsibility and strength	0.799		

FD1	<---	Sense of moral burden	0.819	0.611	0.862
FD2	<---	Sense of moral burden	0.785		
FD3	<---	Sense of moral burden	0.735		
FD4	<---	Sense of moral burden	0.784		

NC1	<---	JAND-CE	0.657	0.562	0.885
NC2	<---	JAND-CE	0.755		
NC3	<---	JAND-CE	0.739		
NC4	<---	JAND-CE	0.759		
NC5	<---	JAND-CE	0.827		
NC6	<---	JAND-CE	0.751		

EC1	<---	ECNQ-CCV	0.915	0.813	0.988
EC2	<---	ECNQ-CCV	0.915		
EC3	<---	ECNQ-CCV	0.912		
EC4	<---	ECNQ-CCV	0.906		
EC5	<---	ECNQ-CCV	0.908		
EC6	<---	ECNQ-CCV	0.891		
EC7	<---	ECNQ-CCV	0.900		
EC8	<---	ECNQ-CCV	0.879		
EC9	<---	ECNQ-CCV	0.883		
EC10	<---	ECNQ-CCV	0.907		
EC11	<---	ECNQ-CCV	0.912		
EC12	<---	ECNQ-CCV	0.889		
EC13	<---	ECNQ-CCV	0.912		
EC14	<---	ECNQ-CCV	0.879		
EC15	<---	ECNQ-CCV	0.910		
EC16	<---	ECNQ-CCV	0.900		
EC17	<---	ECNQ-CCV	0.877		
EC18	<---	ECNQ-CCV	0.911		
EC19	<---	ECNQ-CCV	0.926		

*Note:* DL, moral responsibility and strength; FD, sense of moral burden; EC, ECNQ-CCV, Ethical Conflict Nursing Questionnaire–Critical Care Version; NC, JAND-CE, Chinese version of judgment about nursing decision.

Abbreviations: AVE, average variance extracted; CR, critical rantion.

**Table 5:** MSQ-R-CV discriminant validity tests for each dimension.

	Moral responsibility and strength	Sense of moral burden
Moral responsibility and strength	0.532	
Sense of moral burden	0.433	0.611
Square root of AVE	0.729	1.782

Abbreviation: AVE, average variance extracted.

**Table 6:** Results of descriptive statistics for each dimension and normality test for measurement items.

Dimension	Measured value	M	SD	Skewness	Kurtosis	Total *M*	Total SD
Moral responsibility and strength	DL1	4.116	1.540	−0.615	−0.483	4.131	1.170
	DL2	4.171	1.417	−0.526	−0.509		
	DL3	4.107	1.497	−0.431	−0.731		
	DL4	4.152	1.486	−0.466	−0.640		
	DL5	4.107	1.471	−0.598	−0.485		

Sense of moral burden	FD1	2.982	1.566	0.593	−0.689	2.969	1.298
	FD2	2.951	1.550	0.568	−0.657		
	FD3	2.979	1.493	0.503	−0.602		
	FD4	2.963	1.562	0.609	−0.589		

JAND-CE	NC1	7.364	1.960	−0.774	−1.094	7.652	1.373
	NC2	7.793	1.610	−1.110	−0.234		
	NC3	7.668	1.762	−1.146	−0.256		
	NC4	7.691	1.668	−0.965	−0.656		
	NC5	7.769	1.658	−1.357	0.374		
	NC6	7.627	1.777	−1.085	−0.425		

ECNQ-CCV	EC1	4.582	7.233	1.744	1.641	4.761	6.547
	EC2	4.774	7.401	1.691	1.515		
	EC3	4.902	7.197	1.618	1.326		
	EC4	5.113	7.711	1.597	1.123		
	EC5	4.665	7.113	1.629	1.396		
	EC6	4.896	7.282	1.684	1.586		
	EC7	4.604	6.995	1.667	1.545		
	EC8	4.683	6.884	1.636	1.437		
	EC9	4.527	7.101	1.790	1.991		
	EC10	4.802	7.315	1.696	1.591		
	EC11	4.710	7.221	1.687	1.569		
	EC12	4.683	7.338	1.765	1.897		
	EC13	4.814	7.200	1.629	1.357		
	EC14	4.924	7.315	1.615	1.265		
	EC15	4.698	7.111	1.608	1.334		
	EC16	4.704	6.992	1.586	1.203		
	EC17	4.902	7.136	1.583	1.254		
	EC18	4.738	7.226	1.653	1.499		
	EC19	4.744	7.355	1.687	1.512		

*Note:* DL, moral responsibility and strength; EC, ECNQ-CCV, Ethical Conflict Nursing Questionnaire–Critical Care Version; FD, sense of moral burden; NC, JAND-CE, Chinese version of judgment about nursing decision.

Abbreviations: M, mean; SD, standard deviation.

**Table 7:** Pearson's correlations among the variables (*n* = 328).

Dimension	Moral responsibility and strength	Sense of moral burden	JAND-CE	ECNQ-CCV
Moral responsibility and strength	1.000			
Sense of moral burden	−0.372⁣^∗∗^	1.000		
JAND-CE	0.352⁣^∗∗^	−0.418⁣^∗∗^	1.000	
ECNQ-CCV	−0.386⁣^∗∗^	0.389⁣^∗∗^	−0.364⁣^∗∗^	1.000

*Note:* JAND-CE, Chinese version of judgment about nursing decision.

Abbreviation: ECNQ-CCV, Ethical Conflict Nursing Questionnaire–Critical Care Version.

⁣^∗∗^*p* ≤ 0.01 (two-tailed), significant correlation.

**Table 8:** Model fitness test.

Norm	Reference standard	Results
CMIN/DF	1–3 as excellent and 3–5 as good	1.490
RMSEA	< 0.05 as excellent and < 0.08 as good	0.039
IFI	> 0.9 as excellent and > 0.8 as good	0.978
TLI	> 0.9 as excellent and > 0.8 as good	0.976
CFI	> 0.9 as excellent and > 0.8 as good	0.978

*Note:* CMIN/DF, chi-square degree of freedom ratio.

Abbreviations: CFI, comparative fit index; IFI, incremental fit index; RMSEA, root mean square error of approximation; TLI, Tucker–Lewis index.

**Table 9:** Path hypothesis test results for the structural equation model of factors influencing ethical conflict.

Path	*β*	S.E.	C.R.	*p*	Bootstraps @ 95%	
					LLCI	ULCI
Moral responsibility and strength⟶JAND-CE	0.263	0.076	3.953	⁣^∗∗∗^	0.126	0.393
Sense of moral burden⟶JAND-CE	−0.353	0.075	−5.194	⁣^∗∗∗^	−0.468	−0.225
Moral responsibility and strength⟶ECNQ-CCV	−0.246	0.354	−3.897	⁣^∗∗∗^	−0.329	−0.045
Sense of moral burden⟶ECNQ-CCV	0.232	0.353	3.561	⁣^∗∗∗^	−0.383	−0.112
JAND-CE⟶ECNQ-CCV	−0.183	0.309	−2.887	0.004	0.094	0.377

*Mediating role of JAND-CE*						

*Moral responsibility and strength⟶JAND-CE⟶ECNQ-CCV*						
Direct effect	−0.246	0.411	−3.897	⁣^∗∗∗^	−2.246	−0.646
Indirect effect	−0.048	0.134	−2.007	0.005	−0.608	−0.07
Total effect	−0.294	0.405	−4.071	⁣^∗∗∗^	−2.52	−0.897

*Sense of moral burden⟶JAND-CE⟶ECNQ-CCV*						
Direct effect	0.232	0.433	3.561	0.001	0.487	2.183
Indirect effect	0.065	0.169	2.071	0.007	0.082	0.758
Total effect	0.297	0.424	3.788	⁣^∗∗∗^	0.824	2.493

*Note:* JAND-CE, Chinese version of judgment about nursing decision.

Abbreviations: C.R., critical ratio; ECNQ-CCV, Ethical Conflict Nursing Questionnaire–Critical Care Version; LLCI, lower limit confidence interval; S.E., standard error; ULCI, upper limit confidence interval.

⁣^∗∗∗^*p* < 0.001, significant correlation.

## Data Availability

The data that support the findings of this study are available on request from the corresponding author. The data are not publicly available due to privacy or ethical restrictions.
